# Calcium-/Calmodulin-Dependent Protein Kinase II (CaMKII) Inhibition Induces Learning and Memory Impairment and Apoptosis

**DOI:** 10.1155/2021/4635054

**Published:** 2021-12-23

**Authors:** Jialu Wang, Xiaoxue Xu, Wanying Jia, Dongyi Zhao, Tomasz Boczek, Qinghua Gao, Qianhui Wang, Yu Fu, Miao He, Ruixue Shi, Xin Tong, Meixuan Li, Yu Tong, Dongyu Min, Wuyang Wang, Feng Guo

**Affiliations:** ^1^Department of Pharmaceutical Toxicology, School of Pharmacy, China Medical University, Shenyang 110122, China; ^2^Department of Neurology, The First Hospital of China Medical University, Shenyang 110001, China; ^3^Department of Pharmacy, Chi Feng City Hospital, Chifeng, Inner Mongolia 024000, China; ^4^Department of Molecular Neurochemistry, Medical University of Lodz, 92215, Poland; ^5^Key Laboratory of Ministry of Education for TCM Viscera-State Theory and Applications, Liaoning University of Traditional Chinese Medicine, Shenyang 110032, China; ^6^Jiangsu Province Key Laboratory of Anaesthesiology, Xuzhou Medical University, 209 Tongshan Road, Xuzhou 221002, China

## Abstract

**Objectives:**

Inhibition of calcium-/calmodulin- (CaM-) dependent kinase II (CaMKII) is correlated with epilepsy. However, the specific mechanism that underlies learning and memory impairment and neuronal death by CaMKII inhibition remains unclear.

**Materials and Methods:**

In this study, KN93, a CaMKII inhibitor, was used to investigate the role of CaMKII during epileptogenesis. We first identified differentially expressed genes (DEGs) in primary cultured hippocampal neurons with or without KN93 treatment using RNA-sequencing. Then, the impairment of learning and memory by KN93-induced CaMKII inhibition was assessed using the Morris water maze test. In addition, Western blotting, immunohistochemistry, and TUNEL staining were performed to determine neuronal death, apoptosis, and the relative signaling pathway.

**Results:**

KN93-induced CaMKII inhibition decreased cAMP response element-binding (CREB) protein activity and impaired learning and memory in Wistar and tremor (TRM) rats, an animal model of genetic epilepsy. CaMKII inhibition also induced neuronal death and reactive astrocyte activation in both the Wistar and TRM hippocampi, deregulating mitogen-activated protein kinases. Meanwhile, neuronal death and neuron apoptosis were observed in PC12 and primary cultured hippocampal neurons after exposure to KN93, which was reversed by SP600125, an inhibitor of c-Jun N-terminal kinase (JNK).

**Conclusions:**

CaMKII inhibition caused learning and memory impairment and apoptosis, which might be related to dysregulated JNK signaling.

## 1. Introduction

Epilepsy is a type of chronic neurological disorder characterized by the unnatural, rapid discharge of neurons [1, 2]. The tremor rat (TRM) (tm/tm) has been cultivated in a Kyoto-Wistar colony [3] and is an advantageous model of animal for genetic epilepsy because of the similarity in pathogenesis to the human epileptic circumstance. Our previous studies found that abnormal variations in the CaMKII pathway are involved in epileptogenesis in TRM rats [4–11]. CaMKII is a vital regulator of multiple signaling pathways initiated by calcium signaling. First, CaMKII couples with calcium-bound calmodulin and phosphorylates target substrates in neurons, such as voltage-gated sodium channels, mitogen-activated protein kinases (MAPKs), and the cAMP response element-binding protein (CREB) [12–14]. Additionally, CaMKII is widely distributed in the hippocampus and cortex [15]. Decreased CaMKII levels have been reported in various epileptic models [16–19]. However, the function of CaMKII in epileptogenesis remains unknown.

A series of studies have shown that MAPKs, comprising extracellular signal-regulated kinase1/2 (ERK1/2), p38 MAPK, and c-Jun N-terminal kinase (JNK), are involved in numerous cellular pathways triggered by extracellular apoptotic stimuli, which also play critical roles in cell death and survival [20, 21]. Bioinformatics studies could reveal how the MAPK pathway influences the molecular mechanisms underlying memory loss in temporal lobe epilepsy (TLE) [22].

CREB is a transcription factor, which plays vital physiological and biochemical roles by combining with the conserved sequence of cAMP response elements, mainly involved in the remodeling, learning, and memory of neurons [23]. Additionally, CREB exhibits neuroprotective roles when its expression is upregulated, which increases expression of prosurvival genes [24]. However, changes in MAPK signaling and CREB in epilepsy and their relationship with CaMKII have not been elucidated.

To illustrate the effect of CaMKII in the context of epilepsy, we used KN93, a specific CaMKII inhibitor, in epileptic models *in vitro* and *in vivo*. In the present research, for the first time, our data demonstrate that inhibiting CaMKII contributes to learning and memory impairment and apoptosis associated with dysregulated JNK signaling.

## 2. Materials and Methods

### 2.1. Animals and Drug Administration

Male and female wild-type Wistar and TRM rats (12 weeks old, 50% male and 50% female) were individually housed in a standard environment. All rats were allowed access to water and food ad libitum and housed under controlled circumstances of appropriate temperature with a 12-hour light/dark cycle. The rats were separated into three groups, including, control, low-dosage, and high-dosage groups. Rats in the control group were given an intraventricular bolus of phosphate-buffered saline (PBS) containing DMSO and sulfobutylether-*β*-cyclodextrin (SBE-*β*-CD). Rats in the low-dosage group were administered 9 *μ*g/kg body weight of KN93 (Tocris, Dallas, TX, USA), and those in the high-dosage group were administered 18 *μ*g/kg body weight of KN93. All lateral ventricular injections were performed at a volume of 5.5 *μ*L. In this study, we administered KN93 at a modified concentration compared with the previous dosage chosen for Sprague-Dawley rats on the seventh day (P7) postpartum [25]. We used both low and high doses of KN93 to *in vivo* experiments. The Institutional Animal Care and Use Committee of the China Medical University confirmed the scheme and procedures of the experiment. Animals were processed with KN93 for 24 hours. In the *in vitro* experiments, the incubation of cells was executed with 5 *μ*M KN93 in DMSO for 24 hours. The control group was exposed to DMSO in hippocampal primary cultured neurons and PC12 cells.

### 2.2. Western Blotting

A bicinchoninic acid (BCA) protein assessment kit (Beyotime, Shanghai, China) was employed to quantify extracted protein in the samples. Protein samples were separated using SDS-PAGE. Subsequently, the proteins were transferred onto a PVDF membrane. The membranes were incubated during the night hours at 4°C comprising the following primary antibodies: mouse anti-caspase-3 (1 : 500; Santa Cruz, Dallas, TX, USA), rabbit anti-Bcl-2 (1 : 500; Santa Cruz, Dallas, TX, USA), rabbit anti-cytochrome c (1 : 500; Santa Cruz, Dallas, TX, USA), rabbit anti-NeuN (1 : 5000; Abcam, Cambridge, UK), rabbit anti-GFAP (1 : 500; Santa Cruz, Dallas, TX, USA), rabbit anti-CaMKII (1 : 500; Santa Cruz, Dallas, TX, USA), rabbit anti-phospho-CaMKII (Thr-286; 1 : 500; Santa Cruz, Dallas, TX, USA), rabbit anti-ERK1/2 (1 : 500; Santa Cruz, Dallas, TX, USA), goat anti-phospho-ERK1/2 (Thr202/Tyr204; 1 : 500; Santa Cruz, Dallas, TX, USA), mouse anti-JNK (1 : 500; Santa Cruz, Dallas, TX, USA), mouse anti-phospho-JNK (Thr183/Tyr185; 1 : 500; Santa Cruz, Dallas, TX, USA), rabbit anti-p38 (1 : 500; Santa Cruz, Dallas, TX, USA), rabbit anti-phospho-p38 (Thr180/Tyr182; 1 : 500; Santa Cruz, Dallas, TX, USA), rabbit anti-CREB (1 : 500; Santa Cruz, Dallas, TX, USA), goat anti-phospho-CREB (Ser133; 1 : 500; Santa Cruz, Dallas, TX, USA), and anti-*β*-actin (1 : 1000; Santa Cruz, Dallas, TX, USA). Membranes were then washed multiple times in TBS and incubated by utilizing horseradish peroxidase-conjugated goat anti-mouse IgG (1 : 5000; Santa Cruz, Dallas, Sas) for 1 hour at ambient temperature or horseradish peroxidase-conjugated goat anti-rabbit IgG (1 : 5000; Santa Cruz, Dallas, Sas) for 2 hours, and as a control loading, *β*-actin was employed. An enhanced chemiluminescence (ECL) kit was employed to observe the immune response bands. Similarly, CaMKII was implemented as a loading control for p-CaMKII assessment.

### 2.3. Morris Water Maze Test

Animals were subjected to the Morris water maze, comprising navigation assessments and a probe analysis, for seven consecutive days, as previously described [26]. Briefly, rats were permitted to swim freely for 1 minute without a platform as the experimental baseline (day 0). During training days, a platform was located under the water in the tank for navigation assessments and each rat was tested four times a day with an interval of 60 seconds for spatial acquisition. If the rats could not detect the platform during 60 seconds, they were picked up and located on the platform for the next 60 seconds. The period and path length required for each rat to find the hidden platform were observed in each experiment. On the sixth day, we used exploratory tests to evaluate memory consolidation. In the current experiment, the platform was eliminated from the tank and the rats were permitted to swim freely for 60 seconds. We chose a position that was 180° from the original position of the platform as the new starting position to ensure that the memory capacity for the target position could be reflected in the spatial preference, rather than a specific path of swimming. The frequency of each rat passing through the center of the quadrant and the percentage of time each that rat stayed in the quadrant were observed within 60 seconds. A system of video tracking (Chengdu Taimeng Tech. Co. Ltd., Chengdu, China) was used to record all data.

### 2.4. Cresyl Violet Staining

To assess the morphological modifications in the brain, brain sections were prepared randomly and Nissl bodies were stained with Cresyl violet (CV). The dissolution of Cresyl violet (0.5 g of crystalline acetate powder) was performed in distilled water (500 mL) containing 1.25 mL of glacial acetic acid with a magnetic stirrer at 60°C. Deparaffinized and rehydrated sections were stained by utilizing 0.1% CV staining solution (50–60 minutes) and fixed in neutral gum solution. CV-stained coronal sections were determined using an optical microscope. The morphological characteristics of pyramidal neurons in the hippocampus (CA1 and CA3) and granular cells in the DG area were analyzed. In addition, cell density was evaluated using the ImageJ software.

### 2.5. Immunofluorescence Staining

Frozen rat brain slices were washed with PBS before being blocked with 5% BSA blocking buffer (Solarbio, Beijing, China) at room temperature (45 minutes). The slices were incubated (overnight at 4°C) with glial fibrillary acidic protein (GFAP) rabbit polyclonal antibody (1 : 200; Bioswamp, Wuhan, China). Slices were then washed with PBS and incubated (1 h at room temperature) using DyLight 488, goat anti-rabbit IgG (1 : 200; Abbkine, Wuhan, China). Nuclei were stained with DAPI (1 : 100; Solarbio, Beijing, China). The images were captured using a confocal microscope and were analyzed using ImageJ.

### 2.6. TUNEL Staining

TUNEL staining was conducted following the standard protocols described in the manual of the One-step TUNEL apoptosis assay kit (Beyotime, Jiangsu, China). The apoptotic cells were double immunofluorescent stained for the TDT-mediated dUTP nick end labeling (TUNEL) assay (green) and the nuclear counter stain DAPI (blue).

### 2.7. PC12 Cell Line Culture

The frozen cell seeds were revived at 37°C for 30 min and poured into a Petri dish with culture medium. The morphology of the cells was observed every 6 hours. When the culture dishes appeared confluent, the cell fluid in the culture dishes was discarded. Then, the cells were slowly transferred into preheated serum-free culture medium and washed thrice. Trypsin was added to the media, and the state of the cells was observed. After complete digestion, medium was added to terminate the digestion and the cell suspension was centrifuged. Then, the cell-free supernatant was discarded and 100 *μ*L DMSO (cell cryopreservation solution) was added. Bovine and horse serum suspension (900 *μ*L) was added, and cells were transferred to cryopreservation tubes for gradient cryopreservation.

### 2.8. Primary Hippocampal Neuron Culture

Within two days after birth, newborn Wistar rat pups were anesthetized and decapitated; the brain was quickly removed, soaked in Hank's solution, and stored at 4°C. The hippocampus was dissected rapidly under a microscope ice bath, and the hippocampal tissue was cleaned three times with refrigerated cell culture medium (5 mL DME/F-12; HyClone, Logan, UT, USA). The hippocampal tissue was digested with 2 mL pectinase and gently agitated twice. The cell suspension was removed to repeat the process with pectinase until complete digestion was achieved. After digestion, the cells were evenly mixed with the culture medium from hippocampal neurons and counted for lamination. Half-volume of medium was replaced every other day 24 hours after adhesion of cells. After culturing cells for 7 days, the neuronal state was observed for 9 days and the subsequent experiments were carried out.

### 2.9. Cell Counting Kit 8 Cytotoxicity Test (CCK8 Cytotoxicity Test)

The cell subculture process was repeated, and the cell suspension was transferred to a plate containing 96 wells with 5000–7000 cells per well (note mixing). After adhering to the cell wall, cells were processed with varying concentrations of the drug (0, 0.1, 0.3, 1, 3, 10, 30, and 100) for 24 hours and the final reaction volume was 100 *μ*L. Then, CCK-8 reagent was added at a 1 : 10 ratio and the cells were incubated for 2 hours. The absorbance at 450 nm was evaluated by employing an enzyme-linked immunosorbent assay (ELISA). If the absorbance was not up to the standard, the absorbance was remeasured every 30 minutes. The rate of survival for the cells was evaluated by applying the following equation:

(1) $$\begin{array}{c} \text{Cell survival rate}\left(\%\right)=\frac{\left[A\left(\text{added}\right)-A\left(\text{blank}\right)\right]}{\left[A\left(0\text{ added}\right)-A\left(\text{blank}\right)\right]}\times 100\%. \end{array}$$

### 2.10. RNA Sequencing and Data Analysis

RNA-sequencing was performed by the Wuhan SeqHealth Technology Company. Cells treated with KN93 and the vehicle control (DMSO) were harvested, and the extraction of RNA was carried out by employing TRIzol (Invitrogen, Carlsbad, CA, USA). An RNA library was prepared first, after which a HiSeq X 10 sequencer (Illumina) was used to enrich and sequence products that ranged in size from 200 to 500 bp. Common upregulated or downregulated differentially expressed genes (DEGs) in the selected datasets were extracted with the cutoff criteria of ∣logFC | >0.585 and *p* < 0.05. Biological information was obtained based on biological functional annotation information. The DEGs were merged into a gene list that was submitted to DAVID, a biological information database, for functional annotation. We further conducted gene ontology (GO) enrichment analysis.

### 2.11. Flow Cytometry

In an ice bath, cells were digested with trypsin and then pelleted by centrifugation. The binding buffer (4 mL) was mixed with 36 mL of deionized water, and the binding buffer reagent was diluted 10 times. The cells were washed with 900 *μ*L PBS. The suspension was centrifuged for 5 minutes at 1000 rpm. Following the elimination of the supernatant, the cells were gently agitated with 1000 *μ*L binding buffer and centrifuged for 5 minutes. The above procedure was repeated twice. After centrifugation, the supernatant was removed and the cell suspension treated with 1000 *μ*L binding buffer was retained in each tube. In addition to PI single staining and a blank control group, 5 *μ*L of FITC (green) was added per tube for 10 minutes. Thereafter, PI (5 *μ*L) was added per tube for 1 hour. Then, 300 *μ*L PBS was added to each tube and flow cytometry was performed. Staining and data statistics: late apoptosis of PI and early apoptosis of FITC. Q4: early; Q2: late; Q1: necrotic. The cell injury was generally dominated by Q2 + Q4.

### 2.12. Statistical Analysis

SPSS (version 11.5; SPSS Inc., Chicago, IL, USA) was employed for statistical assessments, and all analysts were blinded to the treatment of each group. All achievements are represented as the mean ± SEM. Student's *t*-test or ANOVA followed by Tukey's test were performed. *p* < 0.05 was set to illustrate the statistical significance.

## 3. Results

### 3.1. Identification of DEGs in Primary Cultured Hippocampal Neurons Regulated by CaMKII Inhibition

**Figure 1:**
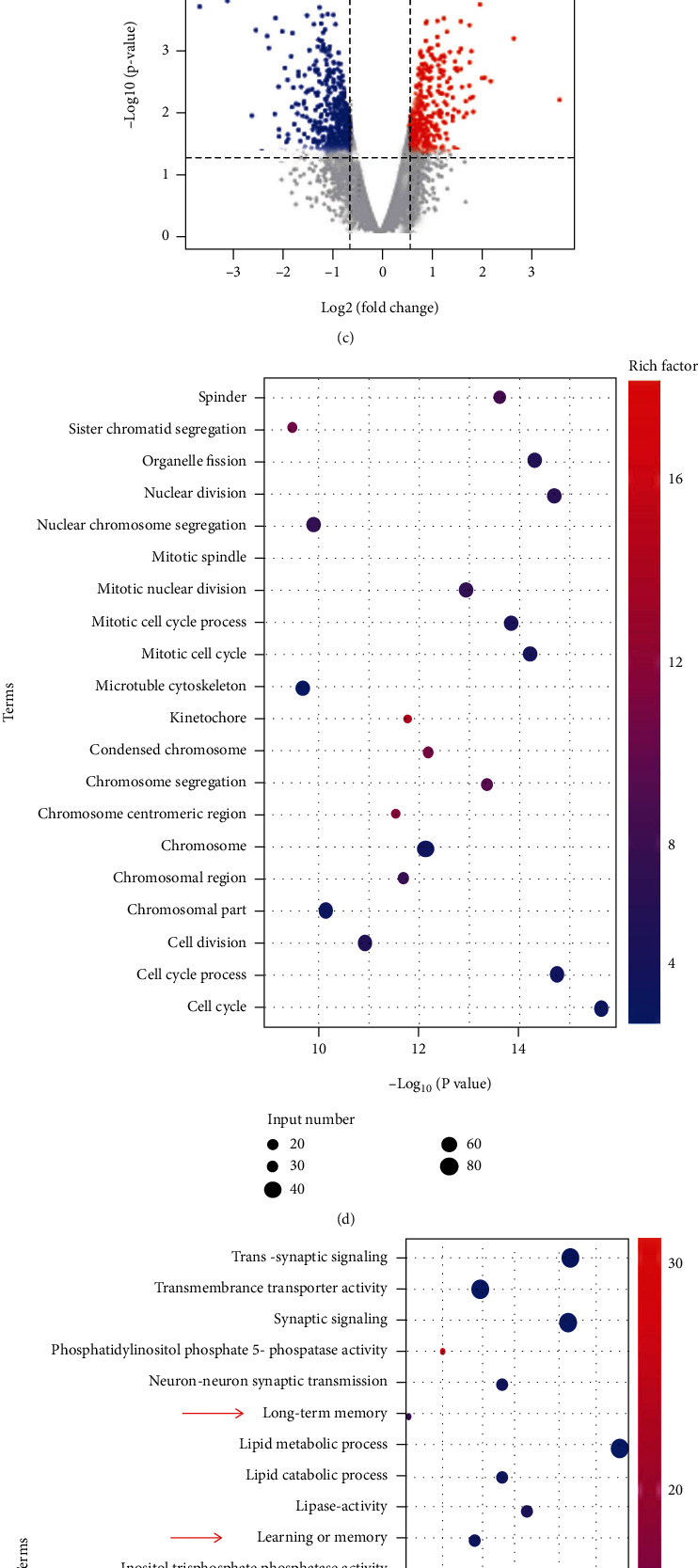
Global identification of target genes in primary cultured hippocampal neurons regulated by CaMKII inhibition. (a) Histogram indicating upregulated and downregulated genes. We observed 404 upregulated genes and 511 downregulated genes in KN93-treated neurons compared with untreated neurons. (b) Difference in gene expression levels in KN93-treated cultured neurons and untreated control groups according to the criteria ∣logFC | >0.585 and *p* < 0.05. The black dots between the two blue lines represent the downregulated genes after CaMKII inhibition, and the red dots on both sides of the blue line represent the upregulated genes. (c) Volcano plot of gene profiles in KN93-treated neurons compared with untreated neurons. Green and red plots represent genes that are abnormally expressed with *p* < 0.05 and ∣log(FC) | >0.585. Red and green plots indicate upregulated and downregulated genes. (d, e) GO analysis indicating differentially expressed genes between KN93-treated neurons and untreated control groups. The genes with higher expression in KN93-treated neurons compared with untreated control groups are shown in (d), while the lower expression genes are shown in (e). The arrows show the genes related to cognition, learning or memory, and long-term memory.

We identified DEGs by RNA sequencing in primary cultured hippocampal neurons of two groups (vehicle for 24 hours, 5 *μ*M KN93 for 24 hours).  Figure 1(a) illustrates 404 upregulated genes and 511 downregulated genes in KN93-treated neurons compared with the untreated controls. Differences in gene expression levels between the KN93-treated and untreated control groups, based on the criteria ∣logFC | >0.585 and *p* < 0.05, are shown in Figure 1(b), and the volcano map is shown in Figure 1(c). The 915 DEGs were subjected to GO enrichment analysis (Figures 1(d) and 1(e)). These downregulated DEGs were related to cognition (GO: 0050890, 22 genes), learning or memory (GO: 0007611, 18 genes), and long-term memory (GO: 0007616, 6 genes), suggesting that CaMKII inhibition can cause learning and memory impairment.

### 3.2. CaMKII Inhibition Induces Learning and Memory Impairment in Both Wistar and TRM Rats

Based on the results of the RNA sequencing analysis, we next checked whether CaMKII inhibition caused learning and memory impairment. According to a previous study, we injected KN93 into the right ventricle of adult TRM rats (*n* = 6) at a dose of 30 *μ*g per kilogram of body weight [25]. However, all TRM rats died following the administration of 30 *μ*g/kg body weight KN93 (*n* = 6). Considering the toxicity, the dose of KN93 was decreased to 18 *μ*g/kg body weight (*n* = 6). As anticipated, the dose of KN93 at 18 *μ*g/kg body weight did not cause death in rats. Thus, we assumed that 18 *μ*g/kg body weight of KN93 may approach the maximum tolerable dose for TRM rats. KN93 was used at two different doses (18 and 9 *μ*g/kg body weight for high and low doses, respectively) in follow-up experiments.

**Figure 2:**
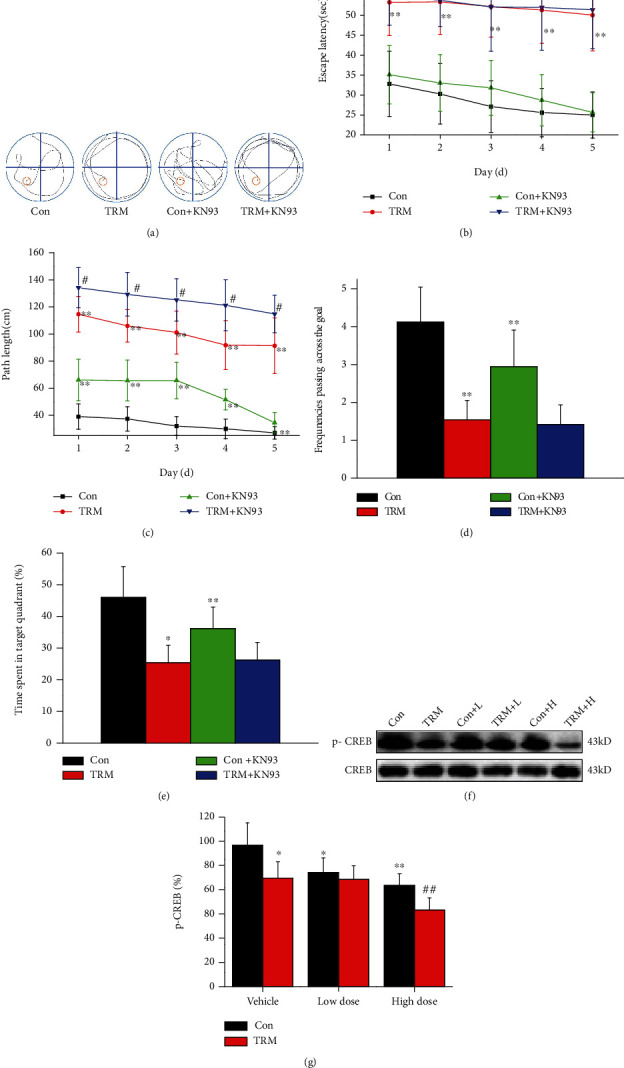
CaMKII inhibition enhances learning and memory impairment in the hippocampal neurons of Wistar and TRM rats. (a) The route trajectory in the hippocampus of Wistar rats, TRM rats, KN93- (high dose) treated Wistar rats, and KN93-treated (high dose) TRM rats. (b) The escape latency in Wistar rats, TRM rats, KN93-treated (high dose) Wistar rats, and KN93-treated (high dose) TRM rats as observed by employing the Morris water maze assessment. (c) The path length in Wistar rats, TRM rats, KN93-treated (high dose) Wistar rats, and KN93-treated (high dose) TRM rats in the navigation test of the Morris water maze. (d, e) Data analysis of the frequencies passing across the goal and the time in the target quadrant in Wistar rats, TRM rats, KN93-treated (high dose) Wistar rats, and KN93-treated (high dose) TRM rats using the Morris water maze. (f, g) Representative protein bands and data analysis of p-CREB proteins in the hippocampus of Wistar rats, TRM rats, KN93-treated (low dose) Wistar rats, KN93-treated (low dose) TRM rats, KN93-treated (high dose) Wistar rats, and KN93-treated (high dose) TRM rats. ^∗^*p* < 0.05, compared with the control Wistar group; ^#^*p* < 0.05, compared with the control TRM group; ^∗∗^*p* < 0.01, compared with the control Wistar group; ^##^*p* < 0.01, compared with the control TRM group.

We then investigated whether KN93 treatment affected learning and memory in Wistar and TRM rats by performing the Morris water maze test (Figure 2). From day to day, the escape latency was higher in the group of TRM compared with the group of Wistar rats, with similar results for path length, which was longer than that in the group of control (*p* < 0.01, *n* = 6, Figures 2(a)–2(c)). The frequency of passing across the goal in the KN93-treated Wistar rats (high dose) was lower than that observed in the Wistar rat control group (*p* < 0.01, *n* = 6, Figure 2(d)). The frequency of passing across the goal in the KN93-treated TRM rats decreased in comparison with that in the group of control (*p* < 0.01, *n* = 6, Figure 2(d)). Similar results were observed for time in the target quadrant (Figure 2(e)).

Previous investigations have shown that histone demethylase PHF2 is able to improve the CREB signaling pathway via regulation of genes related to memory [27]. We next determined the expression level of CREB in Wistar and TRM rats after treatment with of KN93 using Western blotting. The expression of p-CREB in KN93-treated TRM rats was decreased compared with control rats (*p* < 0.05, *n* = 6, Figures 2(f) and 2(g)). Additionally, the expression of p-CREB in Wistar rats after the application of KN93 (high and low dose) was significantly reduced compared with that in control Wistar rats (*p* < 0.01 and *p* < 0.05, *n* = 6, Figures 2(f) and 2(g)). Moreover, the p-CREB levels in TRM rats after the administration of KN93 (high dose) decreased in comparison to control TRM rats (*p* < 0.05, *n* = 6, Figures 2(f) and 2(g)). Thus, CaMKII inhibition by KN93 treatment induced learning and memory impairment in both Wistar and TRM rats.

### 3.3. CaMKII Inhibition Induces Neuronal Death In Vivo and In Vitro

**Figure 3:**
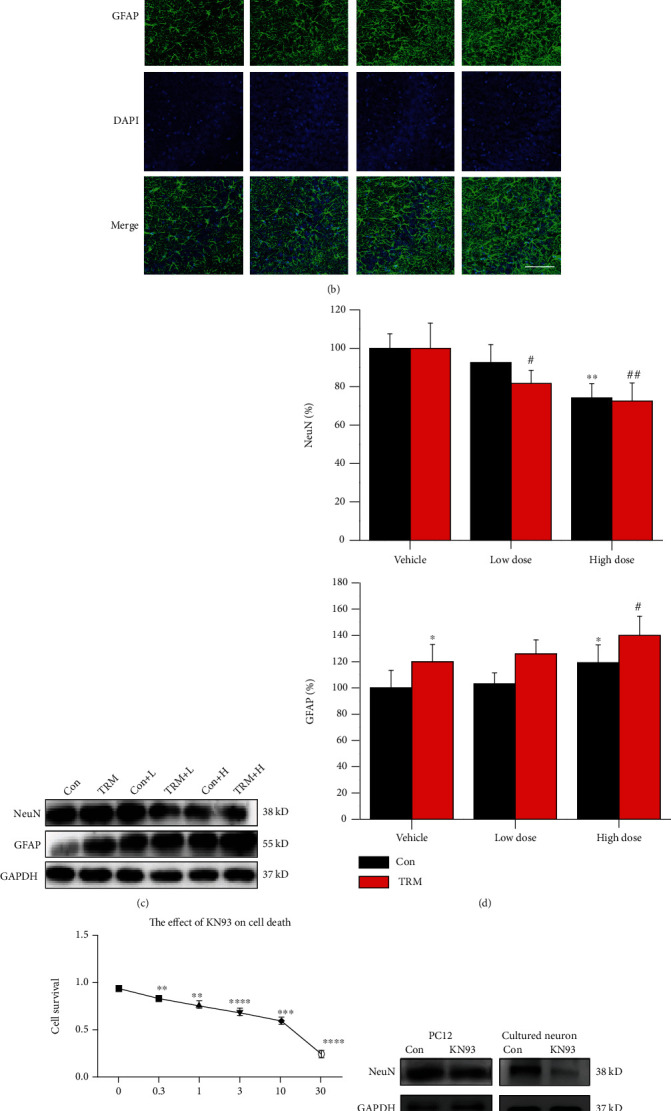
CaMKII inhibition induces neuronal death *in vivo* and *in vitro*. (a) Cresyl violet stain images showing the hippocampi of Wistar rats, TRM rats, KN93-treated (high dose) Wistar rats, and KN93-treated (high dose) TRM rats, including the CA1, CA3, and DG regions. The arrows indicated survived neurons, and the arrowheads indicated dead neurons. Scale bars: 20 *μ*m. (b) Representative images showing the immunofluorescence staining of GFAP in the CA3 regions of the hippocampi of Wistar rats, TRM rats, KN93-treated Wistar rats, and KN93-treated TRM rats. Scale bars: 100 *μ*m. (c, d) Representative protein bands and analysis of data for NeuN and GFAP protein expression in the hippocampi of Wistar rats, TRM rats, KN93-treated (low dose) Wistar rats, KN93-treated (low dose) TRM rats, KN93-treated (high dose) Wistar rats, and KN93-treated (high dose) TRM rats. ^∗^*p* < 0.05, compared with the control Wistar group; ^#^*p* < 0.05, compared with control TRM group; ^∗∗^*p* < 0.01, compared with control Wistar group; ^##^*p* < 0.01, compared with the control TRM group. (e) The concentration-dependent cytotoxicity curve of KN93 on PC12 cells at concentrations of 0.3, 1, 3, 10, and 30 *μ*M. ^∗∗^*p* < 0.01, ^∗∗∗^*p* < 0.001, and ^∗∗∗∗^*p* < 0.0001 compared with controls. (f, g) Expression and localization of NeuN-positive neurons and its data analysis in primary hippocampal neurons of the control group and KN93-treated group. ^∗^*p* < 0.05, compared with the control group; ^∗∗^*p* < 0.01, compared with the control group.

It has been observed in VaD rats that by regulating CaMKII-related signal pathways, apoptosis and inflammation can be inhibited [28]. Thus, we examined whether CaMKII inhibition induced neuronal death in TRM rats. We examined the effects of KN93 using CV staining and astrocyte immunofluorescence staining. Dead cells were recognized morphologically by changes in the size and shape and condensed nuclei. The nuclei of the pyramidal and granule cells with a well-described nuclear membrane and apparently visible nucleoli were identified as live cells. As shown in Figure 3(a), KN93 (high dose) induced death of neurons in the DG, CA3, and CA1 regions of the hippocampal regions of TRM and control Wistar rats. GFAP is a specific marker of astrocytes. As shown in Figure 3(b), KN93 (high dose) induced astrocytes activation in the CA3 regions of the rat hippocampi of four groups. The GFAP immunofluorescence staining of the rest of the regions was shown in supplemental Figure S1. The expression of neuronal marker NeuN and astrocyte marker GFAP was analyzed using Western blotting, as demonstrated in Figure 3(c). The expression of NeuN in both control Wistar and high-dose KN93-treated TRM rats decreased significantly compared with that in both control Wistar and TRM rats (*p* < 0.01, *n* = 6, Figures 3(c) and 3(d)). However, the expression level of GFAP in both control Wistar and high-dose KN93-treated TRM rats increased significantly compared with those detected in control Wistar and TRM rats (*p* < 0.05, *n* = 6, Figures 3(c) and 3(d)). In conclusion, our results indicate that CaMKII inhibition induced neuronal death and reactive astrocyte activation in both Wistar and TRM rat hippocampi.

Furthermore, we determined the concentration-effect curve of KN93 in cells using the CCK8 assay. As shown in Figure 3(e), the cell survival rate decreased as KN93 concentration increased and 25 *μ*mol was calculated as the half-maximal inhibitory concentration (IC_50_) of KN93 in PC12 cells. The protein expression of NeuN in PC12 cells and primary cultivated hippocampal neurons in the KN93-processed group was lesser than that in the group of control (*p* < 0.05 and *p* < 0.01, respectively, *n* = 6, Figures 3(f) and 3(g)). Altogether, CaMKII inhibition by KN93 treatment caused neuronal death *in vitro* and *in vivo*.

### 3.4. CaMKII Inhibition Induces Apoptosis in the Hippocampi of Wistar and TRM Rats

**Figure 4:**
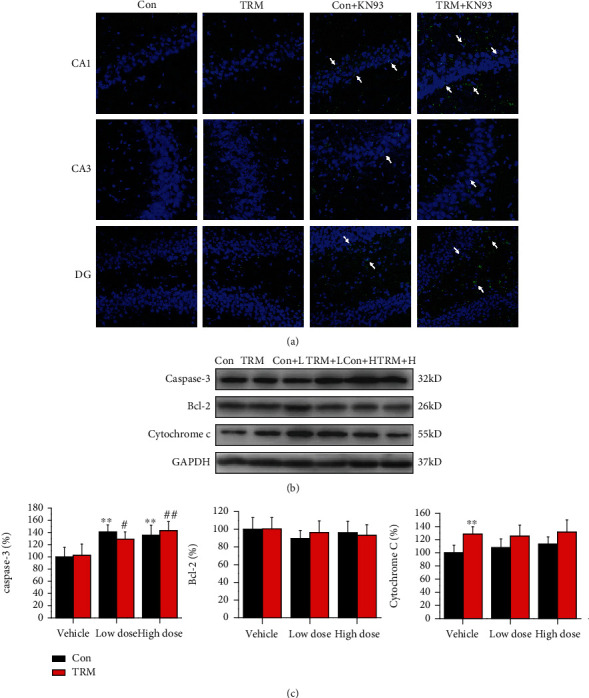
CaMKII inhibition promotes apoptosis in the hippocampi of Wistar and TRM rats. (a) Images indicating TUNEL staining in the hippocampi (CA1, CA3, and DG regions) of Wistar rats, TRM rats, KN93-treated (high dose) Wistar rats, and KN93-treated (high dose) TRM rats. The arrows indicated apoptotic neurons. Scale bars: 100 *μ*m. (b, c) The representative protein bands and data analysis of caspase-3, Bcl-2, and cytochrome c in the hippocampus of Wistar rats, TRM rats, KN93-treated (low dose) Wistar rats, KN93-treated (low dose) TRM rats, KN93-treated (high dose) Wistar rats, and KN93-treated (high dose) TRM rats. ^∗^*p* < 0.05, compared with the control Wistar group; ^#^*p* < 0.05, compared with the control TRM group; ^∗∗^*p* < 0.01, compared with the control Wistar group; ^##^*p* < 0.01, compared with the control TRM group.

We next investigated whether CaMKII inhibition can induce neuronal apoptosis in TRM rats. Surprisingly, we found that the apoptotic cells were showed in the CA1, CA3, and DG regions of both control Wistar and TRM rats after administration of KN93 (high dose) by TUNEL staining in Figure 4(a). However, the apoptotic cells did not appear in the hippocampi of untreated Wistar and TRM rats. Next, we detected the expression of proteins, related to apoptosis (Figure 4(b)). Caspase-3 expression was increased in the hippocampi of Wistar and TRM rats after treatment with high doses of KN93 (all *p* < 0.01, *n* = 6, Figures 4(b) and 4(c)). Additionally, protein expression of cytochrome c in the TRM hippocampus was increased in comparison to that in control Wistar rats (*p* < 0.01, *n* = 6, Figures 4(b) and 4(c)). Thus, CaMKII inhibition by KN93 treatment induced apoptosis in the hippocampal neurons of Wistar and TRM rats.

### 3.5. CaMKII Inhibition Dysregulates MAPK Signaling in Wistar and TRM Rats

**Figure 5:**
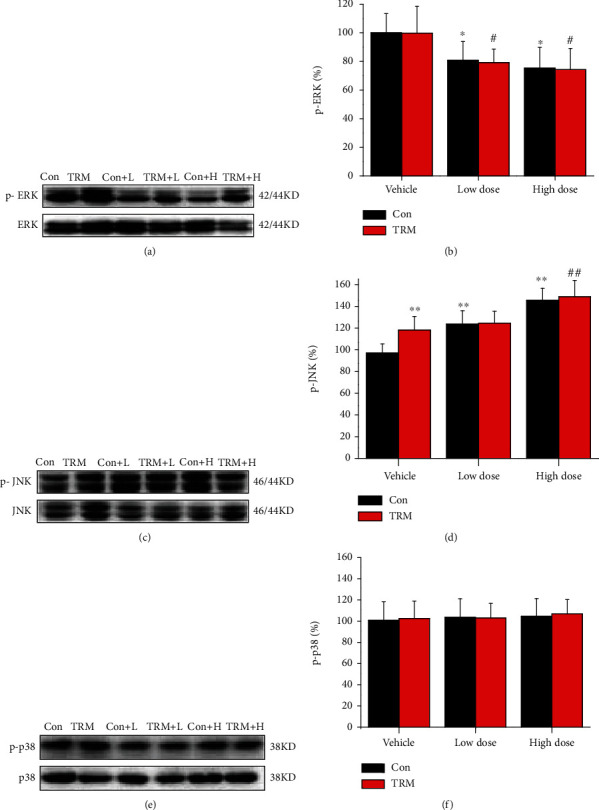
CaMKII inhibition dysregulates MAPKs in Wistar and TRM rats. (a–f) Representative protein bands and analysis of data for p-ERK, p-JNK, and p-p38 proteins in the hippocampi of Wistar rats, TRM rats, KN93-treated (low dose) Wistar rats, KN93-treated (low dose) TRM rats, KN93-treated (high dose) Wistar rats, and KN93-treated (high dose) TRM rats. ^∗^*p* < 0.05, compared with the control Wistar group; ^#^*p* < 0.05, compared with the control TRM group; ^∗∗^*p* < 0.01, compared with the control Wistar group; ^##^*p* < 0.01, compared with the control TRM group.

MAPKs are extremely important in regulating cellular physiological activities, including apoptosis [29, 30]. Thus, we determined MAPK expression levels in Wistar and TRM rats after administration of KN93. We observed that p-ERK expression in TRM rats did not differ compared with that in healthy rats. However, expression levels of p-ERK in the hippocampal neurons of control Wistar rats and TRM rats after treatment with KN93 were substantially decreased compared with those detected in control Wistar and TRM rats (all *p* < 0.01, *n* = 6, Figures 5(a) and 5(b)). Additionally, compared with the control Wistar rats, the expression of p-JNK in TRM rats increased (*p* < 0.01, *n* = 6, Figures 5(c) and 5(d)). The expression of p-JNK in the hippocampal neurons of control Wistar rats after treatment with KN93 (high and low dose) was substantially enhanced compared with that in control Wistar rats (all *p* < 0.01, *n* = 6, Figures 5(c) and 5(d)). Moreover, p-JNK expression was upregulated in TRM rat hippocampi after treatment with KN93 (high dose) compared with that in the untreated TRM group. However, the expression of p-p38 was unaltered in both TRM and Wistar rats after treatment with KN93 (all *p* > 0.05, *n* = 6, Figures 5(e) and 5(f)). In summary, CaMKII inhibition by KN93 treatment dysregulated MAPK signaling in Wistar and TRM rats.

### 3.6. p-JNK Signaling Pathway Participates in Cell Death via CaMKII Inhibition

**Figure 6:**
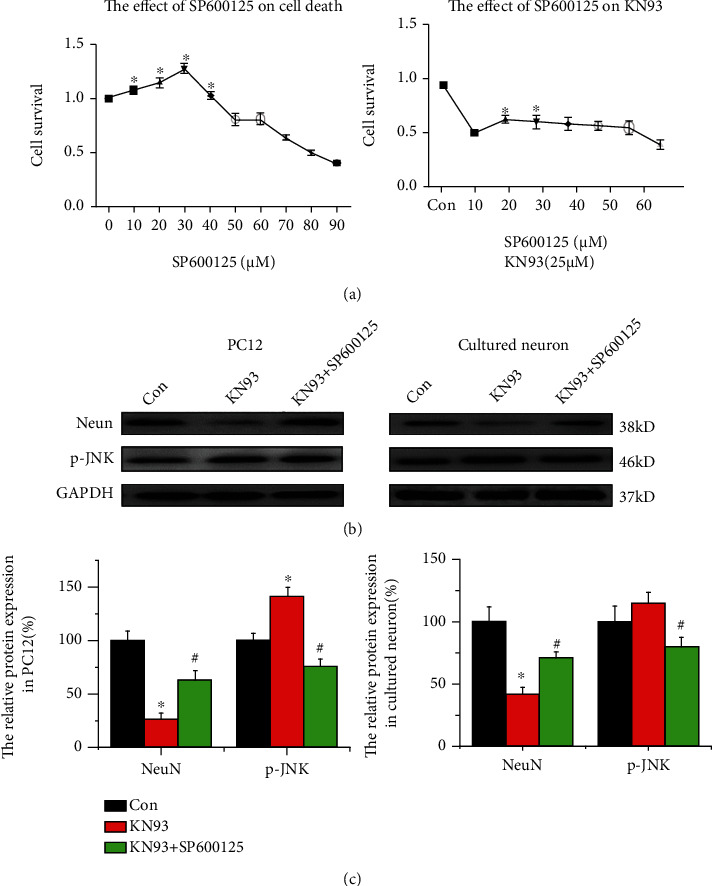
The p-JNK signaling pathway contributes to KN93-induced neuronal death. (a) The concentration-dependent curve of KN93-induced cytotoxicity shows treatment with the 0, 10, 20, 30, and 40 *μ*M p-JNK inhibitor (SP600125) for 24 h after KN93 administration. (b, c) Representative protein bands and analysis of data for p-JNK and NeuN in control groups, KN93 groups, and KN93 + sp600125 groups of PC12 cells and primary hippocampal neurons. ^∗^*p* < 0.05, compared with the control group; ^#^*p* < 0.05, compared with the KN93 group.

Next, we tested whether an ATP-competitive inhibitor of JNK, SP600125, affected KN93-induced cell death *in vitro*. First, the CCK8 assay was used to define the effect of the p-JNK inhibitor (SP600125) on KN93-treated cell death, as shown in Figure 6(a). We found that treatment of 25 *μ*M KN93-treated cells with 10 *μ*M and 20 *μ*M SP600125 enhanced cell survival, compared with the SP600125-untreated group (all *p* < 0.05, *n* = 6, Figure 6(a)). Western blotting results showed that NeuN and p-JNK expression was decreased in the group receiving SP600125 and KN93 expression was decreased in PC12 cells and primary cultured neurons compared with that in the SP600125-untreated group (all *p* < 0.05, *n* = 6, Figures 6(b) and 6(c)). Thus, the signaling pathway of p-JNK takes part in cell death via CaMKII inhibition.

### 3.7. p-JNK Signaling Pathway Contributes to Apoptosis via CaMKII Inhibition

**Figure 7:**
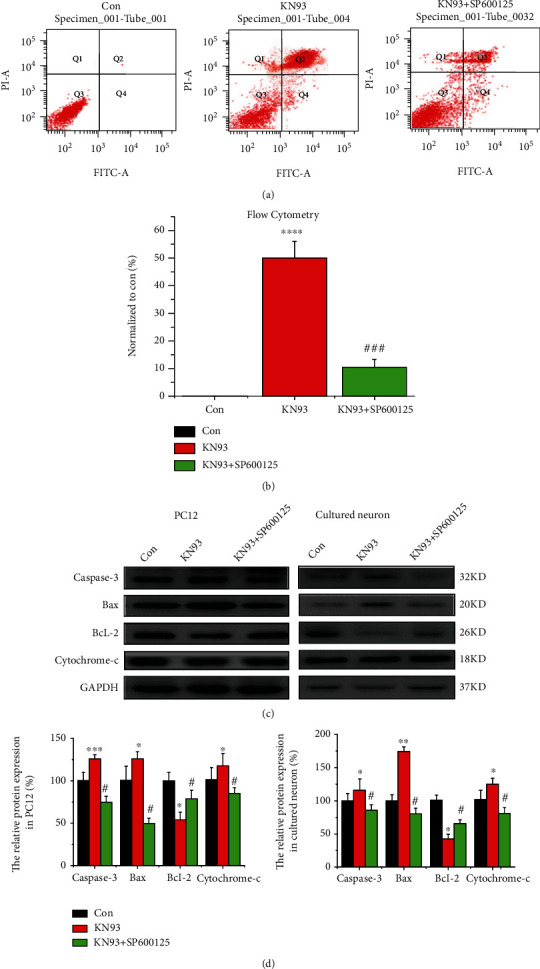
The p-JNK signaling pathway contributes to KN93-induced apoptosis. (a, b) Apoptosis was observed in the control group, KN93 group, and KN93 + sp600125 group of PC12 cells using flow cytometry. ^∗^*p* < 0.05, compared with the control group. (c, d) Representative protein bands and analysis of data for caspase-3, Bcl-2, Bax, and cytochrome c in control groups, KN93 groups, and KN93 + sp600125 groups of PC12 cells and primary hippocampal neurons. ^∗^*p* < 0.05, compared with the control group; ^∗∗^*p* < 0.01, compared with the control group; ^∗∗∗^*p* < 0.001, compared with the control group; ^#^*p* < 0.05, compared with the KN93 group.

We investigated whether p-JNK signaling participates in apoptosis via CaMKII inhibition *in vitro*. The expression of Q2 and Q4 was reduced in the SP600125 and KN93 groups compared with SP600125-untreated cells, indicating that SP600125 reversed KN93-induced apoptosis (*n* = 6, *p* < 0.05, Figures 7(a) and 7(b)). Next, several apoptosis-related proteins have been detected and the representative bands are shown in Figure 7(c). Expression of caspase-3, Bax, and cytochrome c was upregulated, but that of Bcl-2 decreased in both PC12 cells and primary hippocampal neurons treated with KN93, compared with the untreated group (all *p* < 0.05 or *p* < 0.01 or *p* < 0.001, *n* = 6, Figures 7(c) and 7(d)). These data demonstrated that KN93 activated the apoptosis pathway and induced apoptosis 24 hours after administration. Moreover, administration of SP600125 together with KN93 decreased the expression of caspase-3, Bax, and cytochrome c and increased Bcl-2 expression, in both PC12 cells and primary hippocampal neurons, compared with the SP600125-untreated groups (all *p* < 0.05, *n* = 6, Figures 7(c) and 7(d)). Thus, administration of the p-JNK inhibitor SP600125 reversed the changes in these apoptosis-related proteins, indicating that p-JNK signaling participates in apoptosis through CaMKII inhibition.

## 4. Discussions

The aims of our study were at discovering the effect of CaMKII in epilepsy and at demonstrating its relationship with neuronal death. We found that treatment with KN93 for 24 hours induced cell death in normal and TRM rats. The concentration of KN93 used in this study was based on a previous study that used the weight of Sprague-Dawley postnatal day 7 (P7) to determine KN93 dosage [25]. CaMKII is a significant component in the signaling of calcium, and its role is often debated [31]. As a small molecule and an inhibitor of CaMKII, KN93 has been shown to be neuroprotective against excitotoxic insults *in vitro* [32, 33]. Moreover, CaMKII performs a vital task in the survival and excitotoxicity of neurons [34]. In our study, we found that neurotoxicity, but not neuroprotection, was induced *in vivo* and *in vitro* by KN93, suggesting that sustained CaMKII inhibition (24 hours) resulted in toxic effect both *in vitro* and *in vivo*.

CREB phosphorylation is the key to various extracellular signal transduction processes that play roles in the activation of downstream target genes and are involved in the occurrence and development of epilepsy [35]. Previous studies have indicated an elevated expression of CREB in epileptic animal models and patients with epilepsy. The expression of CREB in hippocampal neurons increased remarkably, compared with the control group, and the increase lasted for at least eight weeks in an epileptic pilocarpine mouse model **[**36**]**. Furthermore, p-CREB expression in the temporal lobe neocortex was enhanced in temporal lobe epileptic patients, compared with that in the group of control **[**37**]**. Nevertheless, decreased expression of p-CREB was detected in pentylenetetrazol-kindled rats compared with control rats, indicating a controversial result for p-CREB levels in different epileptic models [38]. In our study, the expression of p-CREB in TRM rats was decreased compared with that in control rats. Additionally, the expression of p-CREB in Wistar and TRM rats after treatment with KN93 was significantly decreased compared with that in control untreated animals, suggesting that p-CREB levels in CaMKII inhibition may be associated with epileptic models.

CREB is mainly involved in neuronal remodeling, learning, and memory in the mature brain [23]. Ca^2+^ influx, CaMKII, and CREB in the hippocampus of chronic epilepsy rat models may be linked to damaged spatial learning and memory [38, 39]. We found that TRM rats exhibited decreased CREB expression and higher escape latency and path length than the control group, suggesting that epileptic rats exhibited cognitive impairment. The path length in the KN93 treatment group increased, and the frequency of passing across the goal and the time in the target quadrant was lower than that in the control Wistar rat group. Thus, our main finding is that CaMKII inhibition induced cognitive impairment in both Wistar and TRM rats.

Furthermore, neuronal apoptosis was observed after the administration of KN93 in *in vivo* and *in vitro* experiments, although a previous study has indicated that apoptotic cell death was not found in SER rats [40]. Caspases have been considered the most important factors involved in programmed cell death in different experimental models of brain impairment, just like epileptic models. These enzymes usually exist in the cytoplasm in latent form and are activated during the later stage of apoptosis. Cytochrome c, when released into the cytoplasm, binds to apoptosis-related factor 1 (Apaf-1) in the presence of dATP to activate the caspase family and induce apoptosis. Caspase-3 is a key mediator of apoptosis in epilepsy [41, 42]. In this study, KN93 treatment led to enhanced levels of activated caspase-3. However, the expression of Bcl-2, an antiapoptotic protein found on the mitochondrial membrane, and cytochrome c was upregulated after the administration of KN93 *in vitro*, indicating that sustained CaMKII inhibition (24 hours) led to neuronal apoptosis.

The MAPK pathway is abundant in the central nervous system (CNS). Extracellular stimuli, such as neurotransmitters, nerve trophic factors, and growth factors, may affect synaptic transmission and cell survival via the MAPK pathway [43–45]. The MAPK family contains ERK1/2, JNK, and p38 MAPK proteins [46]. MAPKs are major factors regulating synaptic excitability, which participate in the regulation of cognitive impairment and epilepsy in animal models and human diseases [47, 48]. A recent study found that the activity of MAPK was substantially greater in pentylenetetrazole-kindled rats in comparison with the group of control [49]. The expression of p-ERK also increased during the spontaneous seizure period after pilocarpine-induced status epilepticus [50]. Blocking ERK1/2 signaling prevented epileptiform behavior in rats [51, 52]. Additionally, several studies have shown that JNK is abnormally activated in epileptic models and is related to cell death [53, 54]. The p38 inhibitor SB203580 decreased pathological damage to the hippocampus and reduced the epileptic frequency [55]. In our study, abnormal expression of MAPKs was observed in TRM rats, but only expression levels of p-JNK in control Wistar and TRM rats were significantly increased after treatment with KN93 compared with those in untreated animals. Moreover, the changes in apoptosis-related proteins in KN93-induced cell death were reversed by administration of the p-JNK inhibitor SP600125, indicating that p-JNK may be a potential therapeutic target for epileptic neuronal death.

## 5. Conclusions

Our study indicates that KN93-induced CaMKII inhibition results in learning and memory impairment, neuronal death, and apoptosis associated with dysregulated p-JNK. Based on the above experimental data, we found that cell death in epilepsy is partly due to the inhibition of CaMKII. The cell death by CaMKII inhibition is related to the duration and concentration of KN93 treatment. However, elucidating the precise underlying mechanism will require further study. These experimental data lay a foundation for understanding the pathogenesis of epilepsy and clarify the toxicological effects and mechanism of CaMKII inhibition by KN93.

## Data Availability

The (data type) data used to support the findings of this study are available from the corresponding author upon request.

## Supplementary Materials

Supplementary Materials Figure S1: CaMKII inhibition induced astrocyte activation in the hippocampus of Wistar and TRM rats. A. Representative images showing the immunofluorescence staining of GFAP in the CA1 and DG regions of the hippocampus of Wistar rats, TRM rats, KN93-treated Wistar rats, and KN93-treated TRM rats. Scale bars: 100 *μ*m. B. The analysis of data showing the relative intensity of GFAP protein expression in the hippocampus of Wistar rats, TRM rats, KN93-treated Wistar rats, and KN93-treated TRM rats. ^∗^*p* < 0.05, compared with the control Wistar group; ^#^*p* < 0.05, compared with control TRM group; ^∗∗^*p* < 0.01, compared with the control Wistar group; ^∗∗∗^*p* < 0.001, compared with the control Wistar group.

## References

[1] Thijs R. D., Surges R., O'Brien T. J., Sander J. W. (2019). Epilepsy in adults. *Lancet*.

[2] Jia W., Song Y., Yang L. (2020). The changes of serum zinc, copper, and selenium levels in epileptic patients: a systematic review and meta-analysis. *Expert Review of Clinical Pharmacology*.

[3] Serikawa T., Yamada J. (1986). Epileptic seizures in rats homozygous for two mutations, zitter and tremor. *The Journal of Heredity*.

[4] Guo F., Zhou P. D., Gao Q. H. (2015). Low-Mg(2+) treatment increases sensitivity of voltage-gated Na(+) channels to Ca(2+)/calmodulin-mediated modulation in cultured hippocampal neurons. *American Journal of Physiology. Cell Physiology*.

[5] Guo F., Xu X., Cai J. (2013). The up-regulation of voltage-gated sodium channels subtypes coincides with an increased sodium current in hippocampal neuronal culture model. *Neurochemistry International*.

[6] Xu X., Guo F., Lv X. (2013). Abnormal changes in voltage-gated sodium channels Na_V_1.1, Na_V_1.2, Na_V_1.3, Na_V_1.6 and in calmodulin/calmodulin-dependent protein kinase II, within the brains of spontaneously epileptic rats and tremor rats. *Brain Research Bulletin*.

[7] Guo F., Yu N., Cai J. Q. (2008). Voltage-gated sodium channel Na_v_1.1, Na_v_1.3 and *β*_1_ subunit were up- regulated in the hippocampus of spontaneously epileptic rat. *Brain Research Bulletin*.

[8] Guan G., Zhao M., Xu X. (2018). Abnormal changes in voltage-gated sodium channels subtypes Na_V_1.1, Na_V_1.2, Na_V_1.3, Na_V_1.6 and CaM/CaMKII pathway in low-grade astrocytoma. *Neuroscience Letters*.

[9] Li J., Yu Z., Xu J. (2018). The effect of Ca2+, lobe-specificity, and CaMKII on CaM binding to NaV1.1. *International Journal of Molecular Sciences*.

[10] Zhao D., Li J., Seehus C. (2018). Bibliometric analysis of recent sodium channel research. *Channels*.

[11] Gao Q., Liu S., Guo F. (2015). Nonylphenol affects myocardial contractility and L-type Ca^2+^ channel currents in a non-monotonic manner via G protein-coupled receptor 30. *Toxicology*.

[12] Zhang G. R., Zhao H., Choi E. M. (2012). CaMKII, MAPK, and CREB are coactivated in identified neurons in a neocortical circuit required for performing visual shape discriminations. *Hippocampus*.

[13] Mollova M. Y., Katus H. A., Backs J. (2015). Regulation of CaMKII signaling in cardiovascular disease. *Frontiers in Pharmacology*.

[14] Shao D., Zhao M., Xu J. (2014). The individual N- and C-lobes of calmodulin tether to the Cav1.2 channel and rescue the channel activity from run-down in ventricular myocytes of guinea-pig heart. *FEBS Letters*.

[15] Takemoto-Kimura S., Suzuki K., Horigane S. I. (2017). Calmodulin kinases: essential regulators in health and disease. *Journal of Neurochemistry*.

[16] Dong Y., Rosenberg H. C. (2004). Prolonged changes in Ca2+/calmodulin-dependent protein kinase II after a brief pentylenetetrazol seizure; potential role in kindling. *Epilepsy Research*.

[17] Davoudi M., Shojaei A., Palizvan M. R., Javan M., Mirnajafi-Zadeh J. (2013). Comparison between standard protocol and a novel window protocol for induction of pentylenetetrazol kindled seizures in the rat. *Epilepsy Research*.

[18] Singleton M. W., Holbert W. H., Ryan M. L., Lee A. T., Kurz J. E., Churn S. B. (2005). Age dependence of pilocarpine-induced status epilepticus and inhibition of CaM kinase II activity in the rat. *Brain Research Developmental Brain Research*.

[19] Blair R. E., Sombati S., Churn S. B., DeLorenzo R. J. (2008). Epileptogenesis causes an _N_ -methyl-d-aspartate receptor/Ca^2+^-dependent decrease in Ca^2+^/calmodulin-dependent protein kinase II activity in a hippocampal neuronal culture model of spontaneous recurrent epileptiform discharges. *European Journal of Pharmacology*.

[20] Karimzadeh F., Modarres Mousavi S. M., Ghadiri T. (2017). The modulatory effect of metabotropic glutamate receptor Type-1*α* on spike-wave discharges in WAG/Rij rats. *Molecular Neurobiology*.

[21] Zhang J., Dai H., Deng Y. (2015). Neonatal chlorpyrifos exposure induces loss of dopaminergic neurons in young adult rats. *Toxicology*.

[22] Liu X., Wu Y., Huang Q., Zou D., Qin W., Chen Z. (2015). Grouping pentylenetetrazol-induced epileptic rats according to memory impairment and microRNA expression profiles in the hippocampus. *PLoS One*.

[23] Ortega-Martinez S. (2015). A new perspective on the role of the CREB family of transcription factors in memory consolidation via adult hippocampal neurogenesis. *Frontiers in Molecular Neuroscience*.

[24] Tan Y. W., Zhang S. J., Hoffmann T., Bading H. (2012). Increasing levels of wild-type CREB up-regulates several activity-regulated inhibitor of death (AID) genes and promotes neuronal survival. *BMC Neuroscience*.

[25] Lu Q., Harris V. A., Sun X., Hou Y., Black S. M. (2013). Ca2+/Calmodulin-Dependent protein kinase II contributes to hypoxic ischemic cell death in neonatal hippocampal slice cultures. *PLoS One*.

[26] Liu M., Chen F., Sha L. (2014). (-)-Epigallocatechin-3-gallate ameliorates learning and memory deficits by adjusting the balance of TrkA/p75NTR signaling in APP/PS1 transgenic mice. *Molecular Neurobiology*.

[27] Kim H. J., Hur S. W., Park J. B. (2019). Histone demethylase PHF2 activates CREB and promotes memory consolidation. *EMBO Reports*.

[28] Jiang H., Ashraf G. M., Liu M. (2021). Tilianin ameliorates cognitive dysfunction and neuronal damage in rats with vascular dementia via p-CaMKII/ERK/CREB and ox-CaMKII-dependent MAPK/NF-kappaB pathways. *Oxidative Medicine and Cellular Longevity*.

[29] Liu H., Nazmun N., Hassan S., Liu X., Yang J. (2020). BRAF mutation and its inhibitors in sarcoma treatment. *Cancer Medicine*.

[30] Shi R., Fu Y., Zhao D., Boczek T., Wang W., Guo F. (2021). Cell death modulation by transient receptor potential melastatin channels TRPM2 and TRPM7 and their underlying molecular mechanisms. *Biochemical Pharmacology*.

[31] Ashpole N. M., Hudmon A. (2011). Excitotoxic neuroprotection and vulnerability with CaMKII inhibition. *Molecular and Cellular Neurosciences*.

[32] Goebel D. J. (2009). Selective blockade of CaMKII-alpha inhibits NMDA-induced caspase-3-dependent cell death but does not arrest PARP-1 activation or loss of plasma membrane selectivity in rat retinal neurons. *Brain Research*.

[33] Vest R. S., O'Leary H., Coultrap S. J., Kindy M. S., Bayer K. U. (2010). Effective Post-insult Neuroprotection by a Novel Ca^2+^/ Calmodulin-dependent Protein Kinase II (CaMKII) Inhibitor. *The Journal of Biological Chemistry*.

[34] Rostas J. A., Hoffman A., Murtha L. A. (2017). Ischaemia- and excitotoxicity-induced CaMKII-mediated neuronal cell death: the relative roles of CaMKII autophosphorylation at T286 and T253. *Neurochemistry International*.

[35] Wang G., Zhu Z., Xu D., Sun L. (2020). Advances in understanding CREB signaling-mediated regulation of the pathogenesis and progression of epilepsy. *Clinical Neurology and Neurosurgery*.

[36] Zhu Y., Li C. S., Wang Y. Y., Zhou S. N. (2015). Change of microRNA-134, CREB and p-CREB expression in epileptic rat. *Asian Pacific Journal of Tropical Medicine*.

[37] Guo J., Wang H., Wang Q., Chen Y., Chen S. (2014). Expression of p-CREB and activity-dependent miR-132 in temporal lobe epilepsy. *International Journal of Clinical and Experimental Medicine*.

[38] Wang P., Wang W. P., Sun-Zhang, Wang H. X., Yan-Lou, Fan Y. H. (2008). Impaired spatial learning related with decreased expression of calcium/calmodulin-dependent protein kinase II_*α*_ and cAMP-response element binding protein in the pentylenetetrazol-kindled rats. *Brain Research*.

[39] Sun W., Feng R., Hu H. (2014). The Ca(2+)-dependent interaction of calpastatin domain L with the C-terminal tail of the Cav1.2 channel. *FEBS Letters*.

[40] Hanaya R., Sasa M., Sugata S. (2010). Hippocampal cell loss and propagation of abnormal discharges accompanied with the expression of tonic convulsion in the spontaneously epileptic rat. *Brain Research*.

[41] Nobili P., Colciaghi F., Finardi A., Zambon S., Locatelli D., Battaglia G. S. (2015). Continuous neurodegeneration and death pathway activation in neurons and glia in an experimental model of severe chronic epilepsy. *Neurobiology of Disease*.

[42] Huang Y. N., Yang L. Y., Wang J. Y., Lai C. C., Chiu C. T., Wang J. Y. (2017). L-Ascorbate protects against methamphetamine-induced neurotoxicity of cortical cells via inhibiting oxidative stress, autophagy, and apoptosis. *Molecular Neurobiology*.

[43] Xia Z., Storm D. R. (2012). Role of signal transduction crosstalk between adenylyl cyclase and MAP kinase in hippocampus-dependent memory. *Learning & Memory*.

[44] RamaRao G., Bhattacharya B. K., Kumar S., Waghmare C. K. (2011). Gene expression and phosphoprotein profile of certain key neuronal signaling proteins following soman intoxication. *Toxicology*.

[45] Gorter J. A., Iyer A., White I. (2014). Hippocampal subregion-specific microRNA expression during epileptogenesis in experimental temporal lobe epilepsy. *Neurobiology of Disease*.

[46] Lai H. C., Chang Q. Y., Hsieh C. L. (2019). Signal transduction pathways of acupuncture for treating some nervous system diseases. *Evidence-based Complementary and Alternative Medicine*.

[47] Kumar V., Zhang M. X., Swank M. W., Kunz J., Wu G. Y. (2005). Regulation of dendritic morphogenesis by Ras-PI3K-Akt-mTOR and Ras-MAPK signaling pathways. *The Journal of Neuroscience*.

[48] Pernice H. F., Schieweck R., Kiebler M. A., Popper B. (2016). mTOR and MAPK: from localized translation control to epilepsy. *BMC Neuroscience*.

[49] Wang X., Huang S., Liu Y., Li D., Dang Y., Yang L. (2021). Effects of ketogenic diet on cognitive function in pentylenetetrazol-kindled rats. *Epilepsy Research*.

[50] Chen J. T., Chen T. G., Chang Y. C., Chen C. Y., Chen R. M. (2016). Roles of NMDARs in maintenance of the mouse cerebrovascular endothelial cell- constructed tight junction barrier. *Toxicology*.

[51] Wu Q., Li Y., Shu Y. (2014). NDEL1 was decreased in the CA3 region but increased in the hippocampal blood vessel network during the spontaneous seizure period after pilocarpine-induced status epilepticus. *Neuroscience*.

[52] Glazova M. V., Nikitina L. S., Hudik K. A. (2015). Inhibition of ERK1/2 signaling prevents epileptiform behavior in rats prone to audiogenic seizures. *Journal of Neurochemistry*.

[53] Solinas G., Becattini B. (2017). JNK at the crossroad of obesity, insulin resistance, and cell stress response. *Molecular metabolism*.

[54] Zhang W., Wang X., Yu M., Li J. A., Meng H. (2018). The c-Jun N-terminal kinase signaling pathway in epilepsy: activation, regulation, and therapeutics. *Journal of Receptor and Signal Transduction Research*.

[55] Zhou X., Chen Q., Huang H. (2020). Inhibition of p38 MAPK regulates epileptic severity by decreasing expression levels of A1R and ENT1. *Molecular Medicine Reports*.

