# Supplementary material for: Calcium-/Calmodulin-Dependent Protein Kinase II (CaMKII) Inhibition Induces Learning and Memory Impairment and Apoptosis
Source: Oxid Med Cell Longev. 2021 Dec 23;2021:4635054. doi: 10.1155/2021/4635054 (PMC8718318; doi:10.1155/2021/4635054)
Supplement: Supplementary Materials — Figure S1: CaMKII inhibition induced astrocyte activation in the hippocampus of Wistar and TRM rats. A. Representative images showing the immunofluorescence staining of GFAP in the CA1 and DG regions of the hippocampus of Wistar rats, TRM rats, KN93-treated Wistar rats, and KN93-treated TRM rats. Scale bars: 100 μm. B. The analysis of data showing the relative intensity of GFAP protein expression in the hippocampus of Wistar rats, TRM rats, KN93-treated Wistar rats, and KN93-treated TRM rats. ∗p < 0.05, compared with the control Wistar group; #p < 0.05, compared with control TRM group; ∗∗p < 0.01, compared with the control Wistar group; ∗∗∗p < 0.001, compared with the control Wistar group. [file 4635054.f1.zip › Dr Wang graphical abstract text (1).docx]

In our study, CaMKII inhibition induced decreased p-CREB and impaired learning and memory in Wistar and Tremor (TRM) rats, an animal model of genetic epilepsy. CaMKII inhibition also induced neuronal death and reactive astrocyte activation in both Wistar and TRM hippocampi, deregulating MAPKs. Meanwhile, neuronal death and apoptosis was observed in PC12 and primary cultured hippocampal neurons after exposure to KN93, which was reversed by the C-Jun N-terminal kinase (JNK) inhibitor SP600125. Our data showed that CaMKII inhibition induced apoptosis by disrupting JNK signaling, which might be a new mechanism of impaired learning and memory in Wistar and TRM rats.
