# Supplementary material for: Calcium-/Calmodulin-Dependent Protein Kinase II (CaMKII) Inhibition Induces Learning and Memory Impairment and Apoptosis
Source: Oxid Med Cell Longev. 2021 Dec 23;2021:4635054. doi: 10.1155/2021/4635054 (PMC8718318; doi:10.1155/2021/4635054)
Supplement: Supplementary Materials — Figure S1: CaMKII inhibition induced astrocyte activation in the hippocampus of Wistar and TRM rats. A. Representative images showing the immunofluorescence staining of GFAP in the CA1 and DG regions of the hippocampus of Wistar rats, TRM rats, KN93-treated Wistar rats, and KN93-treated TRM rats. Scale bars: 100 μm. B. The analysis of data showing the relative intensity of GFAP protein expression in the hippocampus of Wistar rats, TRM rats, KN93-treated Wistar rats, and KN93-treated TRM rats. ∗p < 0.05, compared with the control Wistar group; #p < 0.05, compared with control TRM group; ∗∗p < 0.01, compared with the control Wistar group; ∗∗∗p < 0.001, compared with the control Wistar group. [file 4635054.f1.zip › Figure S1 legend.docx]

**Figure S1. CaMKⅡ inhibition induced astrocyte activation** **in the hippocampus of Wistar and TRM rats.**

A. Representative images showing the immunofluorescence staining of GFAP in the CA1 and DG regions of the hippocampus of Wistar rats, TRM rats, KN93-treated Wistar rats, and KN93-treated TRM rats. Scale bars: 100 μm. B. The analysis of data showing the relative intensity of GFAP protein expression in the hippocampus of Wistar rats, TRM rats, KN93-treated Wistar rats, and KN93-treated TRM rats. * *p<*0.05, compared with control Wistar group; ^#^ *p<*0.05，compared with control TRM group; ** *p<*0.01, compared with control Wistar group; *** *p<*0.001, compared with control Wistar group.
